# Adjusting protein graphs based on graph entropy

**DOI:** 10.1186/1471-2105-15-S15-S6

**Published:** 2014-12-03

**Authors:** Sheng-Lung Peng, Yu-Wei Tsay

**Affiliations:** 1 Department of Computer Science and Information Engineering, National Dong Hwa University, Hualien 974, Taiwan

## Abstract

Measuring protein structural similarity attempts to establish a relationship of equivalence between polymer structures based on their conformations. In several recent studies, researchers have explored protein-graph remodeling, instead of looking a minimum superimposition for pairwise proteins. When graphs are used to represent structured objects, the problem of measuring object similarity become one of computing the similarity between graphs. Graph theory provides an alternative perspective as well as efficiency. Once a protein graph has been created, its structural stability must be verified. Therefore, a criterion is needed to determine if a protein graph can be used for structural comparison. In this paper, we propose a measurement for protein graph remodeling based on graph entropy. We extend the concept of graph entropy to determine whether a graph is suitable for representing a protein. The experimental results suggest that when applied, graph entropy helps a conformational on protein graph modeling. Furthermore, it indirectly contributes to protein structural comparison if a protein graph is solid.

## Background

Graph theory is now widely used in information theory, combinatorial optimization, structural biology, chemical molecule, and many other fields. Graph similarity measuring is a practical approach in various fields. When graphs are used to represent of structured objects, the problem of measuring similarities between objects becomes one of computing similarities between graphs [1]. Protein remodeling is another field wherein multiple-domains within structures are considerably complicated.

It is believed that proteins are important molecules for living organisms. In fact, they are essential parts of organisms and participate in almost every process within cells. A protein contains at least one linear chain of amino acid residues called a *polypeptide*. By various synthesis, *e.g*., biosynthesis and chemical synthesis, a polypeptide is folded into a unique 3-dimensional structure. Usually, the structure of a protein determines its biological function performed in organisms. Knowledge of a protein structure can help us understand biological functions and evolution. Measuring protein similarities according to 3-dimensional structures of proteins provides a valuable tool for evaluating proteins with low sequence similarities when evolutionary relations among proteins cannot be detected by sequence alignment techniques. To perform a structural comparison of molecules, accurate information of two superimposed protein structures must be obtained. However, optimizing these two quantities simultaneously is difficult. Unlike the sequence alignment problem, the structural alignment problem has not even been classified as solvable.

For decades, studies have attempted to define topological relations and notations on protein structures, a schematic description is essentially expected to describe its topology. Mathematical formulations of structural patterns can facilitate the composition in a polypeptide chain. A schematic description has the advantage of simplicity, making the implementation of graph theory as an alternative approach possible [2]. By selectively ignoring protein structural features, it has the potential to detect further homologous relationships based on various geometric methods and motivations.

The structure of a protein can be regarded as a conformation with various local elements (*e.g*., helixes, sheets) and forces (*e.g*., Van der Waal's forces, hydrogen bonds), folding into its specific characteristic and functional structure. With the help of graph transformation, folded polypeptide chains can be represented as a graph using several mapping rules. Proteins contain complex relationships in its polymer: residual reactions, covalent interactions, peptide bonding, and hydrophobic packing are essential parts in structural determination. The intention is to transform a protein structure into a graph. Formally, a *graph transforming system *consists of a set of graph rewrite rules: *L *→ *R*, which *L *is called pattern graph and *R *is called replacement graph [3]. It is the key operation in graph transformation.

### Protein Remodeling

**Figure 1:**
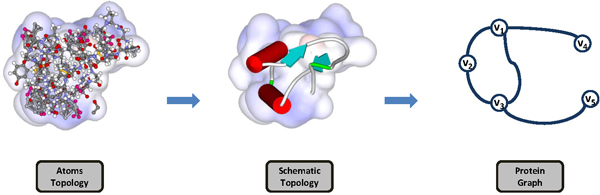
**An overview of protein graph remodeling**.

As mentioned to the protein remodeling, a study reviewed in detail of protein graph (abbreviated as P-graph) description can be found in [4]. Usually, the vertex set of a P-graph can be defined by C*_α _*atoms, residues, side chains, DSSP (the dictionary of protein secondary structures), and SSE (secondary structure elements). For the edge set, it is usually defined by the distance of two vertices with some labels, *e.g*., chemical properties. Figure 1 shows an overview of protein graph remodeling. Table 1 shown an outline of some categories of the protein graph approach to a set of graphs, representing each specific graph rewriting and graph measuring skills. Therefore, it is useful to begin with the summarized common research into the following matters: *geometric relation *and *chemical relation*.

**Table 1:** Recent studies for constructing protein graphs.

**Ref**.	Vertex Set	Edge Set
[20]	*C_α _*atoms	labeled edges
[21]	DSSP	attributed edges
[22]	side chains	defined by interacted energy
[23]	residues	defined by geometrical constraints
[24]	SSE	labeled edges

Proteins have been represented in various ways using different levels of detail. The conformation of protein structure has been shown to be determined geometrically by various constraints [5]. Therefore, the most common method for protein modeling is to reserve its topological relationship in graphs. From the perspective of graph theory, a simplified representation of protein structure aims attention at connectivity patterns. It helps to go into details on interacted relation within a polypeptide folding. In brief, the geometric-based protein modeling is to refine its edges (relations) among vertices (objects), adapting the information from inter-object distances for all pairs of objects.

Comparing with geometric relationship, chemical properties provides a more complicated description in the protein graph model. Amino acid contain various chemical properties, including electrostatic charge, hydrophobicity, bonding type, size and specific functional groups [6]. By giving values to edges and nodes in graph, each different labeled component that varies between the various types of chemical relation.

### Entropy

*Entropy *defines a quantitative equilibrium property within a system and it implies the principle of disorder from the second law of thermodynamics [7]. It is particularly important in describing how energy is applied and transferred in an isolated system. The higher the disorder, the greater the entropy of the system [8]. Similarly, this concept is presented in life. As we known, life is composed of many cells, tissues, and organs from the vital element of protein. Since proteins are biochemical compounds, consisting of one or more polypeptide chains, the arrangement of protein polymers are assumed to be in a compact state, according to its backbone dihedral angles and side chain rotamers. This is called *conformational entropy*. There is considerable evidence to prove that the same observation can be applied to a protein graph model. In such a case, a graph model should also follow the second law of thermodynamics.

For an *n*-object system *G*, assume that each object *i *is associated with a probability *p_i_*. Then the entropy of the system *G *is defined as in Formula 1 [9].

(1) $$I\left(G\right)=\overset{n}{\underset{i=1}{\sum }}-p_{i}\times {\text{log}}_{2}p_{i}$$

In graph theory, the entropy of a graph is usually defined by its degree sequence. For example, we consider the cycle with 4 vertices, *i.e*., *C*_4_. The degree sequence is (2, 2, 2, 2). Thus, the *p_i _*for each vertex *v_i _*is $\frac{2}{8}=0.25$. By definition, *I*(*C*_4_) = −4 × 0.25 × log_2_(0.25) = 2.

## Methods

In this section, we extend the concept of graph entropy to measuring protein graphs. To demonstrate the calculation of graph entropy exemplarily, peptide chains of MHC (Major Histocompatibility Complex) are selected as the materials for examining the utilities of graph entropy.

### Graph entropy

For a given graph *G *= (*V, E*) and two vertices *u *and *v *in *V *, let *d*(*u, v*) denote the length of the shortest path between *u *and *v*. Let *N_k _*(*u*) = {*v* | *d*(*u, v*) = *k*}. In graph theory, *N_k_*(*u*) is called the *k*-distance neighborhood of *u *and is also called the *k*-sphere of *u *[10]. By counting *k*-distance neighbors of *v_i_*, it gives a good account of nodes mutual connectivity in *G*. We define the following formula.

(2) $$f\left(u\right)=\overset{k}{\underset{i=1}{\sum }}\frac{\left|N_{i}\left(u\right)\right|}{n-i+1}$$

In Formula 2, *k *is the longest length for *u *to reach to a vertex, (i.e., *N_k _*(*u*) ≠ ∅ but *N_k+1_*(*u*) = ∅). The idea of our formula makes that every other vertex *v *contributes an impact to the current vertex *u*. In particular, the closer distance between *v *and *u*, the greater the impact of *v*. For simplicity, we let *f*(*V*) = ∑*_v∈V _f*(*v*). Assume that *V *= {*v*_1_*, v*_2_*, . . . , v *}. We define *q_i _*for each *v_i _*as follows.

(3) $$q_{i}=\frac{f\left(v_{i}\right)}{f\left(V\right)}$$

Finally, our modified entropy formula for a graph *G *= (*V, E*) is as follows.

(4) $$I^{\prime }\left(G\right)=\overset{n}{\underset{i=1}{\sum }}-q_{i}\times {\text{log}}_{2}q_{i}$$

For convenience, we consider the graph depicted in Figure 1 as an example which is a P-graph based on small proteins of the plant crambin. This graph is an unlabeled graph corresponding to the protein. The following equations are easy to obtain:

$$\begin{array}{ccc} f\left(v_{1}\right) & = & f\left(v_{3}\right)=\frac{3}{5}+\frac{1}{4}\\ f\left(v_{2}\right) & = & \frac{2}{5}+\frac{2}{4}\\ f\left(v_{3}\right) & = & f\left(v_{5}\right)=\frac{1}{5}+\frac{2}{4}+\frac{1}{3} \end{array}$$

So the entropy of graph depicted in Figure 1 is:

(5) $$\begin{array}{ccc} I^{\prime }\left(G\right) & = & -q\left(v_{1}\right){\text{log}}_{2}q\left(v_{1}\right)-q\left(v_{2}\right){\text{log}}_{2}q\left(v_{2}\right)\\ & & -q\left(v_{3}\right){\text{log}}_{2}q\left(v_{3}\right)-\cdots -q\left(v_{5}\right){\text{log}}_{2}q\left(v_{5}\right)\\ & = & -2q\left(v_{1}\right){\text{log}}_{2}\left(q\left(v_{1}\right)\right)-q\left(v_{2}\right){\text{log}}_{2}\left(q\left(v_{2}\right)\right)\\ & & -2q\left(v_{4}\right){\text{log}}_{2}\left(q\left(v_{4}\right)\right)\\ & = & -2\times 0.3188\times {\text{log}}_{2}\left(0.3188\right)-0.2455\times {\text{log}}_{2}\left(0.2455\right)\\ & & -2\times 0.2214\times {\text{log}}_{2}\left(0.2214\right)\\ & = & 2.4835 \end{array}$$

**Figure 2:**
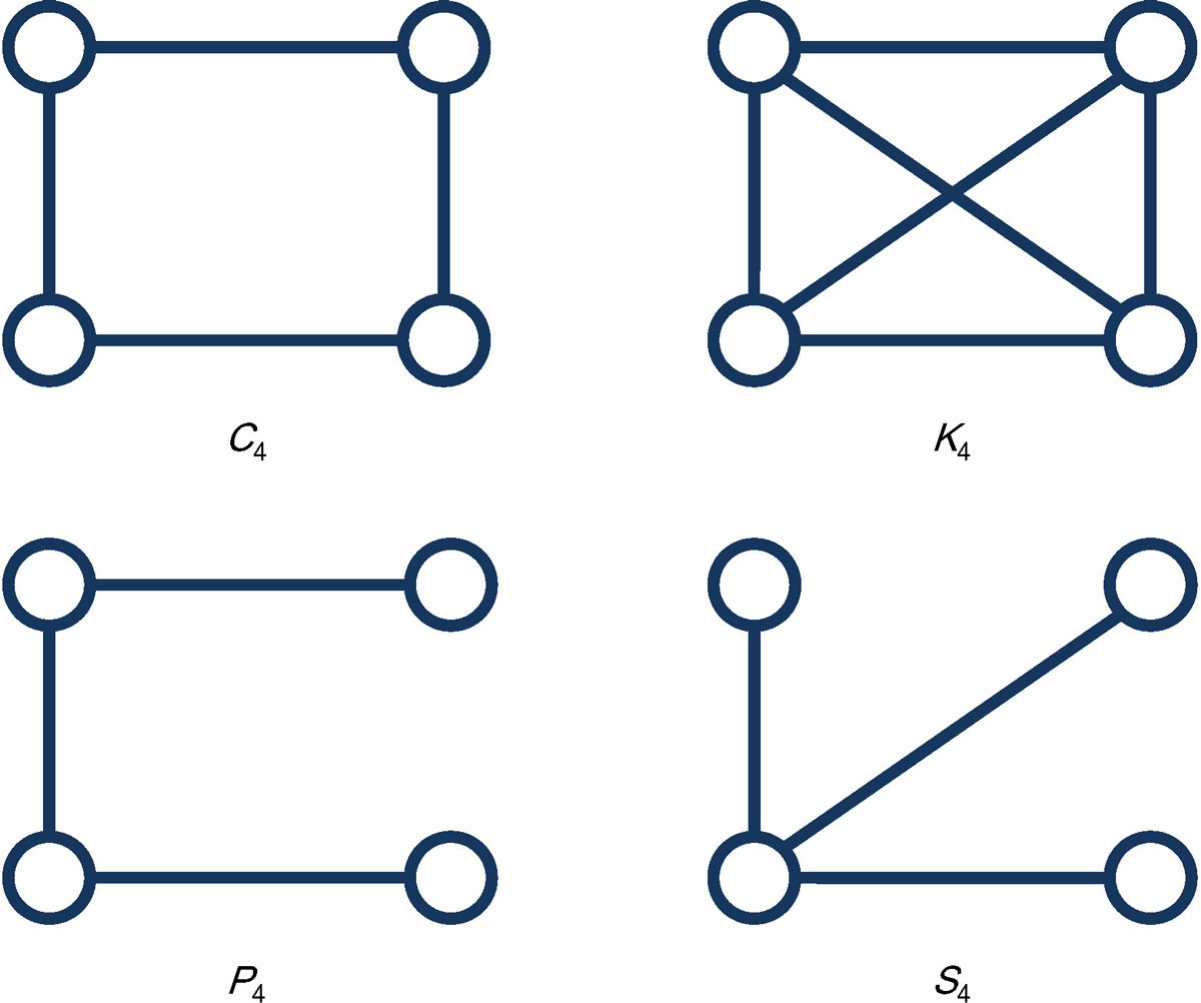
**The four graphs, *C*_4_, *K*_4_, *P*_4_, and *S*_4_**.

Let us consider the four graphs depicted in Figure 2. They are *C*_4_, a cycle of four vertices, *K*_4_, a clique of four vertices, *P*_4_, a path of four vertices, and *S*_4_, a star of four vertices. For *C*_4 _and *K*4, since the four vertices are in the same situation, they have the same probability. Thus *I*(*C*4) = *I′*(*C*_4_) = *I*(*K*_4_) = *I′*(*K*_4_) = log2(4) = 2. However, for *P*_4 _and *S*_4_, we have *I*(*P*_4_) = 1.918 and *I*(*S*_4_) = 1.793. By Formula 4, we have *I′(P*_4_) = 1.894 and *I′*(*S*_4_) = 1.995. The densities of *P*_4 _and *S*_4 _are the same, (*i.e*., 0.5). However, the diameter of *P*_4 _is greater than that of *S*_4_. According to traditional graph entropy, *I*(*S*_4_) <*I*(*P*_4_) *< I*(*K*_4_) = *I*(*C*_4_). However, in our formula, *I′*(*P*4) *< I′*(*S*_4_) *< I′*(*K*_4_) = *I′*(*C*_4_). Intuitively, *S*_4 _is more compact than *P*_4_. Thus, our formula makes a better decision. Note that in graph entropy, the higher entropy of a graph indicates that the graph structure is more stable.

### Edge adjustment

By the definition of *I′*(*G*), its value is not increased monotonously if the density of *G *is increased. Thus, we have the following cases to determine how to adjust the graph. Assume that *G *is the current graph and *I′*(*G*) = *x*. Let *I′*(*G *− *e*) = *y *and *I′*(*G + e*) = *z *where *G *− *e *means that we remove the longest edge from *G *and *G + e *means that we add a shortest non-edge to *G*.

• **Case 1: ***y *= 0 It means that after this edge is removed, *G *is no longer a connected graph.

• **Case 2: ***z > × > y *It means that by adding a new edge, *G *will become more stable.

• **Case 3: ***x > z > y *It means that *G *is stable enough.

• **Case 4: ***y > × > z *It means that by removing an old edge, *G *will become more stable.

**Figure 3:**
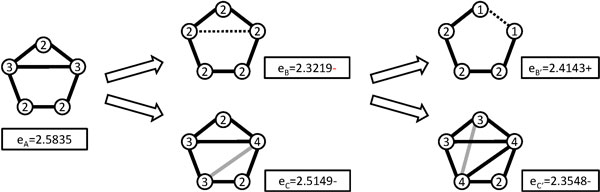
**The effects for increasing and decreasing edges from a graph**.

**Figure 4:**
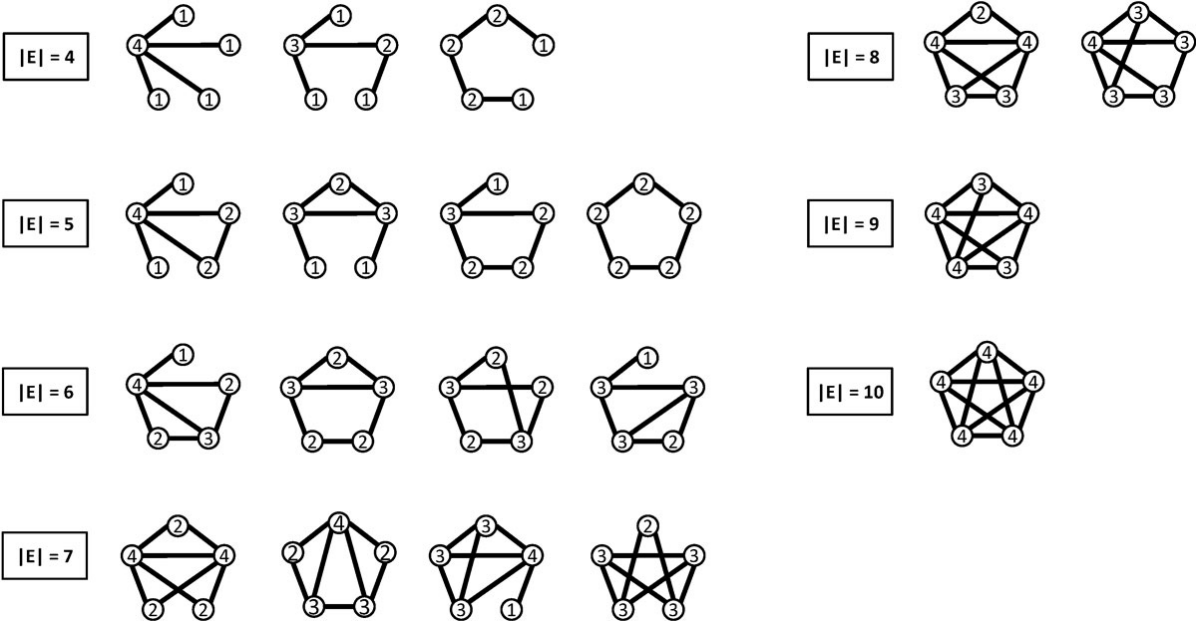
**A set of connected 5-node graphs**.

As illustrated in Figure 3, it shows when edges are added or removed from a graph, their entropy values will be changed. A set of connected 5-node graphs is shown in Figure 4.

### Graph spectra

Given two graphs *G_A _*= (*V_A_, E_A_*) and *G_B _*= (*V_B_, E_B_*), the graph matching problem is to find a one-one mapping *f : V_A _*→ *V_B _*such that if (*u, v*) ∈ *E_A_*, then the possibility of (*f*(*u*), *f *(*v*)) ∈ *E_B _*is as higher as possible. Therefore, numerous attempts have been made on graph similarity to show its efficiency in recent years. In [11], it revealed that the problem of graph matching may be divided into different types depended on their levels. According to the graph scoring, it qualitatively measures a mutual dependence of two objects [12]. Generally, a value of similarity ranges between 0 and 1, from dissimilar to identical.

Occasionally, topologies of graphs are complicated; therefore, one practical way is to symbolize it as matrix, turning graph into numbers and vectors. Since it is hard to determine graph isomorphism, *graph spectra *gives an alternative solution for graph matching. By definition, a spectrum of a finite graph *G *is the spectrum of its adjacency matrix *A_G _*and diagonal degree matrix *D_G_*, whose entries *a_i,j _*and *d_i,j _*can be written as in Formula (6) and Formula (7), respectively. That is, its connected neighbors of eigenvalues together with their multiplicities [13].

(6) $$a_{i,j}=\left\{\begin{array}{cc} 1, & \text{if}\;\left(i,j\right)\in E,\\ 0, & \text{otherwise}\text{.} \end{array}\right)$$

(7) $$d_{i,j}=\left\{\begin{array}{cc} \text{deg}\left(v_{i}\right), & \text{if}\;i=j,\\ 0, & \text{if}\;i\neq j\text{.} \end{array}\right)$$

The *Laplacian spectrum *of *G *is the matrix, *L_G _= D_G _*− *A_G_*, indicating a topological properties and connectedness of the graph. In brief, a graph spectra of *G *can be regarded as a set of *eigenvectors*-- *λ *[14]. Apparently, comparing with the binary relation of graph *G*, the spectrum of *G *tends to improve its information on adjacent relation. We give some examples to describe a graph spectrum transformation. Let *X *be the resulting graph by removing one edge from *K*_4_. Let *Y *be the graph depicted in Figure 1.

(8) $$L_{X}=\left(\begin{array}{ccccc} 3 & -1 & -1 & -1 & 0\\ -1 & 2 & -1 & 0 & 0\\ -1 & -1 & 3 & -1 & 0\\ -1 & 0 & -1 & 2 & 0\\ 0 & 0 & 0 & 0 & 0 \end{array}\right)$$

(9) $$L_{Y}=\left(\begin{array}{ccccc} 3 & -1 & -1 & -1 & 0\\ -1 & 2 & -1 & 0 & 0\\ -1 & -1 & 3 & 0 & -1\\ -1 & 0 & 0 & 1 & 0\\ 0 & 0 & -1 & 0 & 1 \end{array}\right)$$

Obviously, |*V_X_*| = 4 *< |V_Y_*| = 5. Therefore, when spectra are different sizes, the smaller one may be padded with zero values to equalize the size of *G_X _*and *G_Y _*. By definition, the spectra *λ_X _*and *λ_Y _*can be obtained, *i.e*., *λ_X _*= [ 4, 4, 2, 0, 0 ] and *λ_Y _*= [ 4, 4, 1, 1, 0 ]. The similarity between *G_X _*and *G_Y _*can be simply measured by the *Euclidean distance *of *λ_X _*and *λ_Y _*. In this case, the similarity of *G_X _*and *G_Y _*is 1.414.

## Results and discussion

In this experiment, we validated the remodeling function of the P-graph by using extended graph entropy to verify the stability of a given P-graph. For the P-graph construction, please refer to [15]. Thus, we were interested in only the impact of connectivity on protein structural similarities. Various types of MHC were chosen as the material to verify the verification of proposed method: **1HDM**, **1K5N**, **2ENG**, **1VCA**, **1ZXQ**, **1UXW**, **1A2Y**, **3ARD**, **2Q3Z**, and **2CRY**. MHC, as an immune system in most vertebrates, encodes for a small complex cell surface protein. It is also known for HLA (Human Leukocyte Antigen), one of the most intensively studied genes in human [16]. Due to a great diversity of microbes in the environment, MHC genes vary widely its peptide through several mechanisms [17]; this is also the major reason why MHC proteins were selected as materials for this studies.

### P-graph comparison

Let *G *= (*V, E*) be the P-graph after remodeling from the construction proposed by [15]. Vertices of *V *in *G *are created according to the DSSP. Under this metric, a protein secondary structure is represented by a single letter code, H-helix (containing **G**, **H**, and **I**), T-hydrogen turn (containing **T**, **E**, and **B**), or C-coiled (containing only **C**). For controlling one variable in this experiment, let the edge set *E *of *G *be changed from a specific range.

A comparison of MHC proteins is shown in Table 2. In the table, **PID **is the protein identification number in PDB [18]. Since MHC proteins are composed of multiple polypeptide chains, they are multimeric **Domain**. Furthermore, **Dens **means the density in the graph. It is defined as $\frac{2|E|}{|V|\left(|V|-1\right)}$ ranging from 0 to 1. **AVG **indicates the average distance within DSSP vertices. If the distance of *v_i _*and *v_j _*is no greater than AVG, then there is an edge between them. In the table, +*ke *(−*ke*) means that we add (remove) the *k *shortest (longest) possible edges. For example, +1*e *means that we add the edge with the shortest length that is greater than **AVG**. In the table, **NaC **indicates that the resulting graph is not a connected graph.

**Table 2:** A selected proteins with corresponding extended entropies.

PID	− 3e	− 2e	− 1e	AVG	+ 1e	+ 2e	+ 3e
**1HDM**	3.343	3.396	3.563	3.319	3.705	3.845	3.765
Dens	0.357	0.393	0.464	0.524	0.535	0.607	0.643

**1K5N**	4.305	5.545	5.564	4.537	4.614	4.732	3.787
Dens	0.436	0.457	0.475	0.509	0.527	0.564	0.571

**2ENG**	4.000	4.091	4.144	4.212	4.294	4.344	4.480
Dens	0.422	0.444	0.467	0.489	0.511	0.533	0.578

**1VCA**	3.106	3.171	3.254	3.221	3.249	3.467	3.493
Dens	0.381	0.429	0.476	0.524	0.571	0.619	0.667

**1ZXQ**	3.494	3.551	3.641	3.709	3.774	3.712	3.907
Dens	0.429	0.464	0.500	0.535	0.571	0.607	0.643

**1UXW**	5.562	5.563	5.646	5.764	5.855	5.950	6.079
Dens	0.456	0.463	0.478	0.500	0.515	0.529	0.551

**1A2Y**	NaC	NaC	2.414	2.507	2.581	2.512	2.510
Dens	-	-	0.400	0.500	0.600	0.700	0.800

**3ARD**	4.460	4.641	4.698	4.756	4.801	4.860	4.932
Dens	0.424	0.470	0.485	0.500	0.515	0.530	0.554

**2Q3Z**	6.611	6.730	6.775	6.834	6.885	6.996	7.302
Dens	0.474	0.486	0.493	0.503	0.511	0.525	0.547

**2CRY**	NaC	NaC	NaC	NaN	NaN	NaN	NaN
Dens	-	-	-	0.667	1.000	1.000	1.000

The relationship between |*E*| and *I′*(*G*) is as follows. First, when the density in *G *increases, the graph *G *appears to go from sparse to dense. However, its extended entropy does not increase completely with its density. It seems a little anomalous in this appearance. Second, the edge set in protein remodeling issue can be determined from its extended entropy. By definition, the P-graph *G *should be a connected graph. Once the *G *becomes a disconnected graph, we cannot decide its entropy. For example, **1A2Y **is not a connected graph when the density is lower than 0.400. Third, *E *appears to be considerably related to *V *in graph entropy. Consider the P-graph **2CRY **as another example. If a protein remodeling function adapts a specific value on the basis of its geometrical edge, then it might be an error to assume a fixed value as a criterion. This is an essential fact to stress. It is worth pointing out that the construction of a P-graph is limited by *V *.

### P-graph verification

To validate the previous assumptions, a method for protein structural comparison is adapted to measure its similarity. Graph spectra gives an alternative solution to graph matching. It is a set of relational parameters, consisting of a characteristic polynomial and eigenvectors of its adjacency matrix or Laplacian matrix. Graph spectra quantitatively provide graph information, *e.g*., structure, topology, connectivity [19]. In Table 3 we list the results of protein structure remodeling matters. The field **Old **shows a remodeling based on the specific value of edge length, and **New **indicates that the edges in *G *are adjusted by extended entropy. The value in each entry is the distance of the two spectra. If our method obtains a better result in the comparison, then we simply mark "+" to denote a better result; otherwise, it is marked "=" (not bad) or "−" (worse). Table 4 shows the CATH codes for the selected macromolecules. In summary, the extended entropy determines a better conformational graph from protein structure remodeling.

**Table 3:** A comparison of protein structure remodelings.

PID		1K5N	2CRY	1VCA	2Q3Z	1ZXQ	1A21	2ENG	1UXW	1A2Y	3ARD
**1HDM**	Old	7.93	23.36	15.68	24.01	13.74	6.54	12.57	7.92	5.75	8.27
	New	7.75	21.12	13.87	23.67	12.11	5.64	11.03	7.25	5.41	7.79
	Result	+	+	+	+	+	+	+	+	+	+

**1K5N**	Old	·	26.58	19.55	26.91	18.02	14.65	17.69	20.44	18.41	25.72
	New	·	23.70	17.39	21.13	15.99	12.83	15.84	17.17	16.94	23.64
	Result	·	+	+	+	+	+	+	+	+	+

**2CRY**	Old	·	·	14.87	12.33	17.13	14.39	15.62	19.33	6.81	18.42
	New	·	·	12.91	34.10	14.92	12.45	17.54	19.35	5.17	19.63
	Result	·	·	+	−	+	+	−	=	+	−

**1VCA**	Old	·	·	·	17.71	5.39	4.83	7.75	11.42	5.45	12.80
	New	·	·	·	29.68	4.47	3.21	6.82	10.07	4.83	11.67
	Result	·	·	·	-	+	+	+	+	+	+

**2Q3Z**	Old New	·	·	·	·	27.57 26.31	29.30 28.35	23.46 21.11	25.45 24.52	38.30 36.72	24.14 23.00
	Result	·	·	·	·	+	+	+	+	+	+

**1ZXQ**	Old New	·	·	·	·	·	3.98 3.41	7.96 7.49	10.52 9.67	6.21 6.53	9.14 8.87
	Result	·	·	·	·	·	+	+	+	−	+

**1A21**	Old	·	·	·	·	·	·	6.24	12.76	7.37	12.85
	New	·	·	·	·	·	·	5.41	11.38	6.91	11.06
	Result	·	·	·	·	·	·	+	+	+	+

**2ENG**	Old	·	·	·	·	·	·	·	4.65	11.42	14.19
	New	·	·	·	·	·	·	·	4.17	10.39	13.82
	Result	·	·	·	·	·	·	·	+	+	+

**1UXW**	Old	·	·	·	·	·	·	·	·	16.24	5.71
	New	·	·	·	·	·	·	·	·	15.41	3.45
	Result	·	·	·	·	·	·	·	·	+	+

**1A2Y**	Old	·	·	·	·	·	·	·	·	·	12.24
	New	·	·	·	·	·	·	·	·	·	11.41
	Result	·	·	·	·	·	·	·	·	·	+

**Table 4:** CATH codes for the selected macromolecules.

PID	Domain	C	A	T	H	S	O	L	I	D
1HDM	A2	2	60	40	10	152	1	1	1	1
	B2	2	60	40	10	137	1	2	1	1
1K5N	A2	2	60	40	9	1	1	1	1	1
2ENG	A2	2	40	40	10	1	1	1	1	1
1VCA	A1	2	60	40	10	135	1	1	1	1
	A2	2	60	40	10	62	1	1	1	1
1ZXQ	A1	2	40	40	10	123	1	1	1	1
	A2	2	40	40	10	121	2	1	1	1
1UXW	A2	2	60	40	10	9	1	1	1	1
	B1	2	60	40	10	3	1	1	1	1
1A2Y	A1	2	60	40	10	8	2	2	1	1
	B1	2	60	40	10	36	2	1	1	1
3ARD	C1	2	60	40	10	18	2	3	2	1
	D1	2	60	40	10	21	4	12	1	1

### Program and environment

The procedure for computing the extended entropy for a P-graph was implemented and has been tested with the MHC PDB dataset. The environment was running under 2 Ghz PC with 512 MB of main memory with Linux-2.6.11-1.1369. The implementation was written using Bash-3.00.16(1) and Octave-3.0.0.

## Conclusion

In this paper, we proposed a measurement to determine graph stability for protein structure remodeling based on graph entropy. Our modified entropy validation shows a positive result for protein structural comparison. This graph-based approach offers a practical concept to support protein structural alignment.

## Competing interests

The authors declare that they have no competing interests.

## Declarations

Publication charges for this article have been funded by the authors.

This article has been published as part of *BMC Bioinformatics *Volume 15 Supplement 15, 2014: Proceedings of the 2013 International Conference on Intelligent Computing (ICIC 2013). The full contents of the supplement are available online at http://www.biomedcentral.com/bmcbioinformatics/supplements/15/S15.
